# Case Report: Safety and Efficacy of Enfortumab Vedotin in a Patient With Metastatic Urothelial Carcinoma Undergoing Peritoneal Dialysis 

**DOI:** 10.3389/fonc.2022.892793

**Published:** 2022-05-25

**Authors:** Kaylyn R. Collette, Zin W. Myint, Saurabh V. Parasramka, Carleton S. Ellis

**Affiliations:** ^1^Department of Pharmacy, University of Kentucky, Lexington, KY, United States; ^2^Markey Cancer Center, University of Kentucky, Lexington, KY, United States; ^3^Department of Internal Medicine, Division of Medical Oncology, University of Kentucky, Lexington, KY, United States; ^4^Department of Medical Oncology, Taylor Regional Hospital, Campbellsville, KY, United States

**Keywords:** peritoneal dialysis, enfortumab vedotin, antibody–drug conjugate, metastatic urothelial carcinoma, end-stage renal disease

## Abstract

The clinical management of metastatic urothelial carcinoma has significantly evolved with the emergence of monoclonal antibodies and antibody-drug conjugates (ADCs). Enfortumab vedotin (EV) was granted approval by the FDA in 2021 for patients with locally advanced or metastatic urothelial carcinoma who have received prior immunotherapy and platinum-containing chemotherapy. Little to no data exist for the use of EV in patients with concurrent end-stage renal disease (ESRD) using either hemodialysis or peritoneal dialysis (PD). Here, we present the case of a patient with metastatic urothelial carcinoma on PD who failed multiple lines of treatment but demonstrated an impressive response to EV without significant toxicity. We discuss the possible impact of peritoneal dialysis on the pharmacokinetics of ADCs and the potential for safe administration based on known pharmacokinetic data.

## Introduction

Regional or distant metastases are present in approximately 12–15% of patients with urothelial carcinoma at the time of diagnosis. Despite high initial reponse rates to chemotherapy, 5-year survival rates are less than 20% (1). However, the treatment landscape for metastatic urothelial cancer has been rapidly evolving in recent years. New therapies such as checkpoint inhibitors and antibody–drug conjugates (ADCs) have emerged for relapsed and refractory disease and have significantly improved progression-free survival and overall survival.

Enfortumab vedotin (EV) is a monoclonal antibody–drug conjugate directed against nectin-4, a protein highly expressed in urothelial carcinoma. The monoclonal antibody is conjugated with monomethyl auristatin E (MMAE), a cytotoxic component that leads to microtubule disruption and cell death (2). In July 2021, the FDA approved EV for patients with advanced bladder cancer who have previously received a programmed death receptor-1 (PD-1) or programmed death-ligand 1 (PD-L1) inhibitor and platinum-containing chemotherapy or are ineligible for cisplatin-containing chemotherapy and have previously received one or more prior lines of therapy based on the results of the EV-201 trial (3). Of the 89 patients in the original study, only two had severely decreased renal function, and neither required dialysis. There were no significant differences in the exposure (AUC) of EV and MMAE in patients with mild, moderate, or severe renal impairment compared with patients with normal renal function. Unfortunately, there is currently little to no data exploring the use of EV in patients with end-stage renal disease (ESRD) on either hemodialysis or peritoneal dialysis (PD). The effect of end-stage renal disease with or without dialysis on the pharmacokinetics of EV or unconjugated MMAE is unknown (2).

Here, we present the case of a patient with metastatic urothelial carcinoma on PD who failed multiple lines of treatment but demonstrated response to EV without significant toxicity.

## Case Presentation

A 68-year-old gentleman with a past medical history of significant for membranous glomerulonephritis, which led to ESRD on PD, initially presented to an outside hospital in January 2021 with gross hematuria and dyspnea. On a CT scan, he was found to have right-sided hydronephrosis with a possible bladder tumor. He was also noted to be severely anemic with a hemoglobin of 5.9. A subsequent CT chest/abdomen/pelvis showed multiple intraluminal lesions concerning for a multifocal bladder neoplasm and non-specific pulmonary nodules. He underwent partial transurethral resection of the bladder tumor in February 2021, and the pathology was consistent with high-grade muscle invasive urothelial carcinoma. His ECOG was 1.

**Figure 1:**
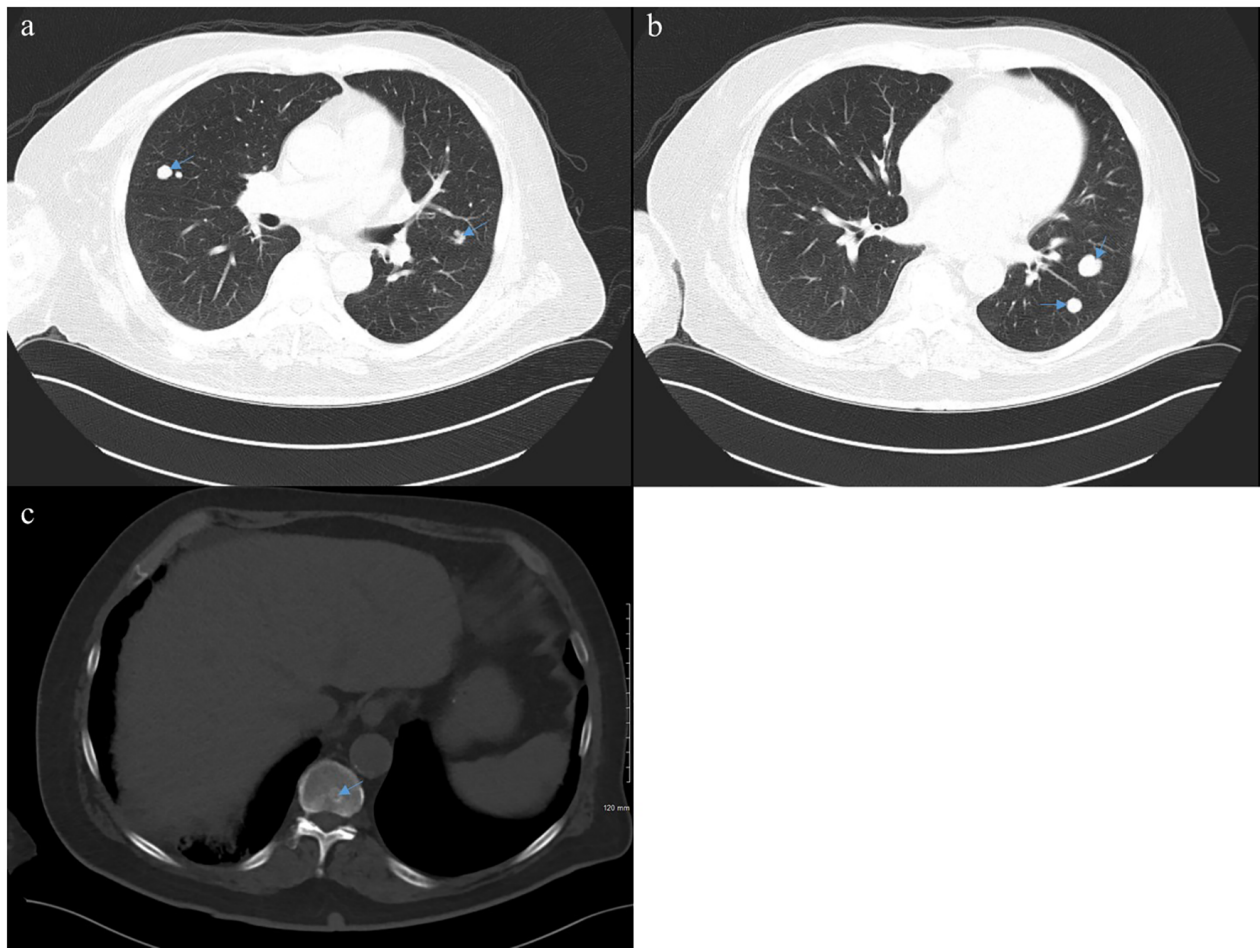
Computed tomography (CT) of the chest, abdomen, and pelvis from April 2021 demonstrating bilateral pulmonary nodules **(A, B)** and an L2 lesion **(C)** consistent with metastatic disease.

**Figure 2:**
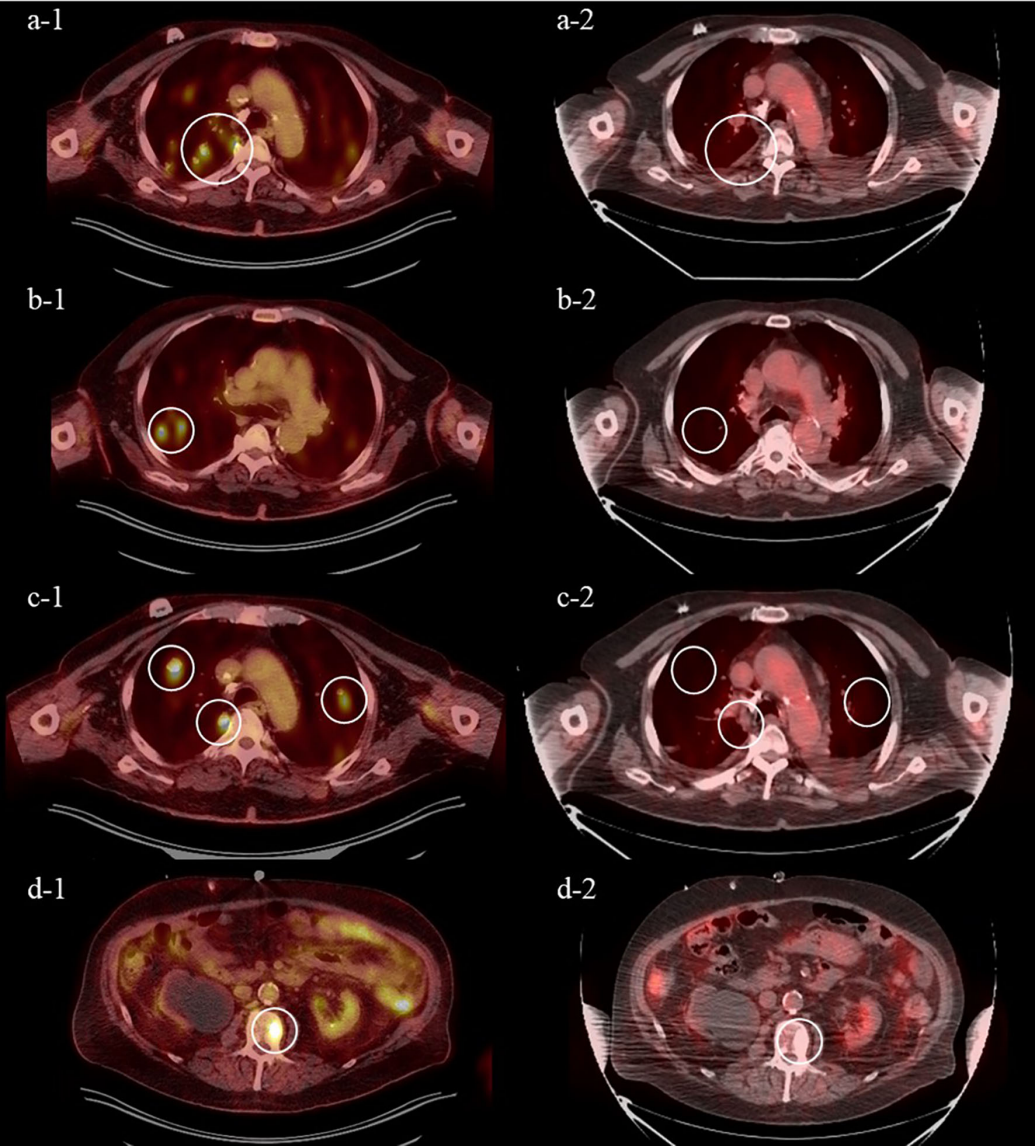
Positron emission tomography (PET) from August 2021 after 4 cycles of pembrolizumab demonstrating increase in size and number of FDG-avid pulmonary nodules **(A-1–C-1)** and FDG-avid sclerotic L2 lesion **(D-1)** and from November 2021 after 3 cycles of enfortumab vedotin demonstrating near complete response of FDG-avid pulmonary nodules **(A-2–C-2)** and FDG-avid L2 lesion **(D-2)**.

At the outside hospital the patient was deemed not a candidate for cisplatin due to his PD, so a radical cystectomy was planned without neoadjuvant chemotherapy. Due to the nonspecific pulmonary nodules concerning for metastatic disease on prior imaging, a restaging CT chest/abdomen/pelvis was performed, and unfortunately, it revealed new and enlarging bilateral pulmonary nodules consistent with pulmonary metastatic disease and a sclerotic L2 vertebral body osseous lesion consistent with bone metastatic disease (**Figure 1**). With surgery no longer an option, the patient received carboplatin (dose reduced to 75 mg/m^2^) and full dose gemcitabine (1,000 mg/m^2^) with growth factor support in May 2021. This was complicated by a prolonged hospitalization secondary to neutropenic fever from *Escherichia coli* sepsis, from which he ultimately recovered. Next-generation sequencing through Caris Life Sciences was performed, which demonstrated a high tumor mutation burden (TMB) of 17, and pathogenic variant BRCA1 mutations at Exon 20 pT1777fs and Exon 3 p.C39G without any identified FGFR alterations. Because of the concern of further myelosuppression and infection, and his noted high TMB, he was subsequently switched to immunotherapy with pembrolizumab 200 mg every 3 weeks as second-line therapy. He received 4 cycles of pembrolizumab from May 2021 to August 2021, which he tolerated well. Unfortunately, a follow-up PET CT scan in August 2021 showed progression in the lungs with numerous bilateral hypermetabolic solid pulmonary nodules that were increased in number and size and raised concern for malignant pleural effusion (**Figure 2**).

**Figure 3:**
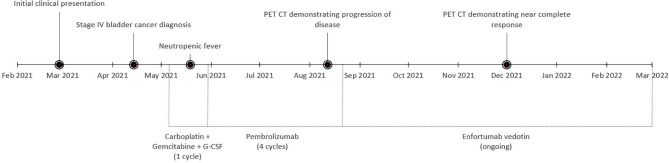
Timeline of events across the treatment course of the patient.

The patient was initiated on EV at 1.25 mg/kg (full dose) on days 1, 8, and 15 of a 28-day cycle in August 2021 as third-line therapy. He tolerated the treatment well without major side effects, except for a grade 2 rash treated with topical and oral corticosteroids and diphenhydramine. A follow-up PET CT scan demonstrated a near complete response after 3 cycles of EV. The previous effusions were smaller and no longer PET-avid, and the previously seen multiple pulmonary nodules and sclerotic L2 lesions were no longer visible (**Figure 3**). At the time of writing, he remains on this therapy.

## Discussion

As far as we are aware, there has been no data published on the efficacy or safety of EV in metastatic urothelial carcinoma patients undergoing PD for end-stage renal disease. We report the first case of the safe administration of this antibody–drug conjugate in a dialysis patient. This case demonstrates the clinical utility and safety of EV in PD patients.

Peritoneal dialysis is one of several modalities used in patients with ESRD. Approximately 12% of dialysis patients utilize PD as renal replacement therapy, highlighting the need for more literature on the safe use of chemotherapy and immunotherapy in this patient population. Patients using PD experience an altered elimination pathway, with pharmacokinetic analyses demonstrating prolonged half-lives, elevated plasma concentrations, and higher AUCs of administered drugs (4). Consequently, incorrect dosing can lead to potentially life-threatening complications and toxicities. It has been demonstrated that correct dosing of chemotherapy based on pharmacokinetic parameters results in better survival outcomes, further highlighting the need to establish clear guidance on appropriate dosing and tolerability of anti-cancer therapy for PD patients (5).

Metastatic urothelial carcinoma is an aggressive malignancy with a high potential for relapsed and refractory disease (6). Despite the contentious nature of the disease, it is typically sensitive to traditional chemotherapy. First-line systemic therapy for locally advanced or metastatic disease includes cisplatin and gemcitabine (7). Gemcitabine generally does not require dose adjustments in PD as it is rapidly converted intracellularly to an inactive metabolite, 2’2’-difluorodeoxycytidine (dFdU) after administration (8). Conversely, cisplatin is primarily renally eliminated. Although nephrotoxicity is not a limitation with PD, patients remain at risk of toxicities such as anemia and neuropathy at supratherapeutic doses. Thus, cisplatin should be administered at 50% of the original dose in PD patients (9). However, current guidelines suggest that patients with renal impairment or other comorbidities should consider carboplatin-based chemotherapy as first-line therapy to minimize toxicities associated with cisplatin therapy (7). Evidence for carboplatin in PD is limited to small studies and case reports. A pharmacokinetic study published by Heijns et al. in 2008 examined a patient on PD while on weekly and three-weekly pacliataxel/carboplatin for recurrent ovarian cancer (10). In this study patient, 14–20% of the carboplatin dose was cleared by PD, while the kidneys cleared 6–8%. Based on these findings, it is generally recommended that patients receive 25% of the calculated carboplatin dose for patients on PD three times daily—guidance implemented in this patient (11, 12).

Patients who relapse on traditional chemotherapy may proceed to monoclonal antibody therapy with checkpoint inhibitors or the ADC EV in the second- and third-line settings (7). Similar to chemotherapy, the guidance for monoclonal antibody administration in PD is limited to case reports and single patient pharmacokinetic studies. Monoclonal antibodies have a small volume of distribution following administration, and distribution in the interstitial space is determined by diffusion and antibody binding followed by intracellular degradation (13). Monoclonal antibodies are too large to be effectively eliminated by dialysis filters, thus the main route of elimination is intracellular catabolism or receptor-mediated endocytosis (13). There have been several case reports published on the use of anti-PD-1 inhibitor therapies, pembrolizumab and nivolumab, in dialysis patients (13–15). However, PD patients comprise only about 3% of the study population examined. Regardless of the small sample size, checkpoint inhibitors without dose adjustment appear to have a similar incidence and severity of adverse events to that seen in the general population.

In addition to anti-PD-1 inhibitor monoclonal antibodies, trastuzumab, an anti-HER-2 monoclonal antibody, has demonstrated limited safety evidence in patients using PD. A recent case report published by Kokkali et al. in 2021 demonstrated the efficacy and safety of trastuzumab in a patient on four-time daily PD (16). The patient received 4 cycles of chemotherapy with IV trastuzumab at standard dosing (8 mg/kg loading dose, followed by 6 mg/kg every 3 weeks), followed by 18 cycles of single-agent trastuzumab. The patient demonstrated adequate exposure to trastuzumab and did not experience any notable toxicities throughout the treatment course.

While there is a limited amount of literature addressing the safety of chemotherapy and immunotherapy in PD patients, there is a particularly severe lack of data on ADC therapy in patients on dialysis. Our case highlights the possible safety and efficacy of EV in a PD patient with metastatic urothelial carcinoma. Further pharmacokinetic studies are warranted to evaluate the efficacy and safety of these agents in patients using renal replacement therapy.

## Data Availability Statement

The original contributions presented in the study are included in the article/supplementary material. Further inquiries can be directed to the corresponding author.

## Ethics Statement

Ethical review and approval was not required for the study on human participants in accordance with the local legislation and institutional requirements. The patients/participants provided their written informed consent to participate in this study.

## Author Contributions

KC wrote the first draft of the manuscript and CE did the major revisions. ZM and SP provided radiographic images and manuscript revisions. All authors listed have made a substantial, direct, and intellectual contribution to the work and approved it for publication.

## Conflict of Interest

The authors declare that the research was conducted in the absence of any commercial or financial relationships that could be construed as a potential conflict of interest.

## Publisher’s Note

All claims expressed in this article are solely those of the authors and do not necessarily represent those of their affiliated organizations, or those of the publisher, the editors and the reviewers. Any product that may be evaluated in this article, or claim that may be made by its manufacturer, is not guaranteed or endorsed by the publisher.
